# Prevalence and Associated Factors of Sexual Dysfunction in Patients With Inflammatory Bowel Disease

**DOI:** 10.3389/fendo.2022.881485

**Published:** 2022-04-22

**Authors:** Jinzhi Zhang, Jiao Nie, Min Zou, Qishan Zeng, Yue Feng, Zhenyi Luo, Huatian Gan

**Affiliations:** ^1^ Department of Geriatrics and National Clinical Research Center for Geriatrics, West China Hospital, Sichuan University, Chengdu, China; ^2^ Laboratory of Inflammatory Bowel Disease, The Center for Inflammatory Bowel Disease, Clinical Institute of Inflammation and Immunology, Frontiers Science Center for Disease-Related Molecular Network, West China Hospital, Sichuan University, Chengdu, China

**Keywords:** inflammatory bowel disease, sexual dysfunction, erectile dysfunction, Crohn’s disease, ulcerative colitis

## Abstract

**Background:**

Sexual dysfunction (SD) in patients who suffer from inflammatory bowel disease (IBD) has not attracted widespread attention, and thus research studies are scarce.

**Objective:**

This study aims to evaluate the rates of SD in IBD compared with healthy individuals and elucidate the associated factors.

**Methods:**

In this cross-sectional study, the Female Sexual Function Index (FSFI) and the simplified version of the International Index of Erectile Function (IIEF-5) were filled by IBD patients, as well as healthy control individuals.

**Results:**

A total of 208 IBD patients, including 133 with Crohn’s disease (CD) and 75 with ulcerative colitis (UC), and 190 healthy individuals filled out the questionnaires. In women, SD rates were 61.9% in the patients with IBD vs. 24.4% in the healthy controls (*p* < 0.01). In men, the rates of erectile dysfunction (ED) were 43.5% in the patients with IBD vs. 12.5% in the healthy controls (*p* < 0.01). Anxiety (OR, 3.092; 95%CI: 1.033-9.252, *p* = 0.044) and active perianal disease (OR, 4.481; 95%CI: 1.055-19.029, *p* = 0.042) were independent risk factors for SD in female IBD patients. age (OR, 1.050; 95%CI: 1.007-1.095, *p* = 0.022), depression (OR, 5.763; 95%CI: 1.864-17.821, *p* = 0.002) and active perianal disease (OR, 7.117; 95%CI: 1.747-28.983, *p* = 0.006) were independent risk factors for ED in male patients.

**Conclusions:**

In the IBD patients, 62% of women reported having SD, and 44% of men reported having ED. These higher rates, as compared to the healthy controls, are mostly driven by active perianal disease and psychological factors.

## Introduction

Inflammatory bowel disease (IBD) mainly encompasses a group of individuals that have Crohn’s disease (CD) or ulcerative colitis (UC) (1). Since the middle of the 20th century, the incidence of IBD in the Western world has reached as high as 0.5% of the total population. This affects more than 1 million people in the United States and roughly 2.5 million people in Europe (2). It is estimated that there will be more than 1.5 million IBD patients in China by the year 2025 (3).

Sexual dysfunction (SD) is a significant health burden characterized by a disturbance in sexual desire and psychophysiological changes in the sexual response cycle, resulting in marked distress and interpersonal difficulty (4). Friedman and Knowles *et al.* reported that the prevalence of SD in male and female IBD patients ranged from 44% to 53.9% and 40% to 66%, respectively (5, 6). For female IBD patients, SD mainly manifests as decreased sexual desire, orgasm, and sexual arousal disorder, while for men, the most common symptom is erectile dysfunction (ED) (7, 8).

However, SD in IBD patients has not attracted widespread attention, and relevant research studies are scarce. As far as we know, there are no studies on the sexual function of both male and female IBD patients in China. Therefore, this study aims to evaluate the prevalence of SD in IBD patients as compared with healthy controls. Furthermore, we aim to look for factors associated with SD in these patients.

## Methods

In this cross-sectional study, all data were reported in IBD units from one China tertiary care university hospital (West China Hospital). All IBD patients were enrolled from February to October 2021 in the inpatient unit. After appropriate training, investigators guide participants to fill in questionnaires. All questionnaires were anonymous, and data was kept anonymous by the use of codes. In separate documentation using the same code, a physician reported the disease activity score. CD patients were assessed by the Crohn’s Disease Activity Index (9), and Improved Mayo Score for UC (10). Disease characteristics were obtained from the patient’s electronic medical file such as IBD subtype, disease duration, active perianal disease, previous surgery, presence of a stoma, and medication, etc. During this same period, healthy controls at the same hospital were invited to participate following the same protocol as the IBD patient cohort. The “Strengthening the Reporting of Observational Studies in Epidemiology” guidelines were applied (11).

### Inclusion and Exclusion Criteria

The following guidelines were used to determine the patient eligibility for inclusion: (1) over 18 years of age, with a stable sexual partner for ≥3 months; (2) IBD diagnosed by “Chinese Consensus on Diagnosis and Treatment of Inflammatory Bowel Disease” (12); (3) data was collected only on the first visit even when there were multiple visits for the same patient; (4) ability to fill in and understand the questionnaire; (5) sign informed consent and participate voluntarily.

The following guidelines were used to exclude patients: (1) those who couldn’t understand or fill out the questionnaire themselves and/or those with mental disorders; (2) diagnosis of SD before an IBD diagnosis; (3) SD compounded by other diseases: cardiovascular and cerebrovascular diseases, lung diseases, liver and kidney dysfunction, and neoplastic diseases, etc.

### Assessment of Basic Characteristics

The questionnaire included socio-demographic and other primary patient data: age, gender, weight, height, smoking history, alcohol consumption, educational background, marital status, employment status, etc.

### Assessment of Anxiety and Depression

This study used the Chinese-validated version of the Hospital Anxiety Depression Scale (HADS) to evaluate psychological function in patients. The HADS has good internal and test-retest reliability and is divided into two subscales: anxiety and depression. If the individual scores for the two subscales are ≥ 8 points, respectively, then the patient is defined as having anxiety or depression (13).

### Assessment of Sexual Function

This study used the Chinese-validated version of the Female Sexual Function Index (FSFI) and a simplified version of the International Index of Erectile Function (IIEF-5) to evaluate SD within female and male patients. The FSFI includes such parameters as desire, arousal, lubrication, orgasm, satisfaction, and pain. The optimal cutoff score of the FSFI is 26.55, which means that a score at or below this value is defined as female SD (14). The IIEF-5 consists of five questions, including vaginal penetration, maintenance of erection, completion of intercourse, sexual satisfaction, and self-confidence of the patient’s sexual arousal and maintenance of an erection. A total score of < 22 is defined as ED (15).

### Outcome

The primary objective of this study was to compare the prevalence of SD between IBD patients and healthy controls. The secondary aim was to find factors associated with SD in patients with IBD.

### Statistical Analyses

Data are presented as median (interquartile range (IQR)) or frequencies, as required. Chi-squared or Student’s *t*-test were used to compare baseline characteristics and scores between groups. Comparison of continuous domain sub-scores and overall scores was performed through a paired Student’s *t*-test on exact age-matched pairs. We performed univariate factor logistic regression analysis on eligible SD risk factors in IBD patients. The event of interest was defined as the occurrence of SD. In univariate logistic regression, factors with *p*-values < 0.20 were contained in the complete model of multivariate logistic regression. Manual stepwise elimination was used to find the most suitable model that is independent of SD factors. The results are expressed as odds ratio with confidence interval values. We used two-sided statistical tests for all analyses. The level of significance used was *p* < 0.05. Statistical analyses were performed using SPSS (v. 24, SPSS, Inc., Chicago, IL, USA).

## Results

### Participant Characteristics

Simultaneously, 228 IBD patients and 190 healthy controls were requested to participate during the study period. Of these patients, 208 (91.2%) completed the questionnaire, while 20 refused. There was no difference in socio-demographic and disease characteristics between the 20 patients who did not complete the questionnaire and the 208 patients who did (**Supplementary Table 1**). The basic characteristics of the participants are shown in **Table 1**. There were 84 female IBD patients (median (IQR) age of 36 (20-67) years old), 78 healthy female controls (median (IQR) age of 37 (20-64) years old), 124 male IBD patients (median (IQR) age of 36 (20-64) years old), and 112 healthy male controls (median (IQR) age of 37 (20-68) years old) available for analysis. IBD and controls were comparable except for BMI, with healthy controls having a higher BMI than the IBD patient group.

**Table 1:** Participants characteristics.

	Female patients	Female controls	*P* value	Male patients	Male controls	*P* value
	*n* = 84	*n* = 78		*n* = 124	*n* = 112	
Age, years			.694			.971
< 41	59 (70.2)	54 (69.2)		85 (68.5)	75 (67.0)	
41-59	23 (27.4)	20 (25.6)		35 (28.2)	33 (29.5)	
> 59	2 (2.4)	4 (5.1)		4 (3.2)	4 (3.6)	
Body mass index, kg/m^2^			.007**			.000**
< 18.5	33 (39.3)	16 (20.5)		26 (21.0)	2 (1.8)	
18.5 – 25	47 (56.0)	55 (70.5)		89 (71.8)	66 (58.9)	
25 – 30	2 (2.4)	7 (9.0)		9 (8.3)	35 (31.3)	
> 30	2 (2.4)	0		0	9 (8.0)	
Education, *n* (%)			.976			.836
Basic level	7 (8.3)	6 (7.1)		9 (7.3)	6 (5.4)	
Medium level	32 (38.1)	29 (37.2)		36 (29.0)	33 (29.5)	
High level	45 (53.6)	43 (55.1)		79 (63.7)	73 (65.2)	
Relationship status, *n* (%)			.248			.161
In a relationship	25 (29.8)	17 (21.8)		34 (27.4)	22 (19.6)	
Married	59 (70.2)	61 (78.2)		90 (72.6)	90 (80.4)	
Active smoker, *n* (%)	2 (2.4)	2 (2.6)	1.000	41 (33.1)	50 (44.6)	.068
Active Drinking, *n* (%)	6 (7.1)	6 (7.7)	.894	26 (21.0)	31 (27.7)	.229
Profession, *n* (%)			.406			.263
Workers	8 (9.5)	7 (9.0)		13 (10.5)	17 (15.2)	
Farmers and herdsmen	11 (13.1)	8 (10.3)		20 (16.1)	11 (9.8)	
Staff	20 (23.8)	28 (35.9)		49 (39.5)	52 (46.4)	
Self-employed	45 (53.6)	35 (43.8)		42 (33.9)	32 (28.6)	
Address, *n* (%)			.168			.054
City	46 (54.8)	51 (65.4)		69 (55.6)	76 (67.9)	
Countryside	38 (45.2)	27 (34.6)		55 (44.4)	36 (32.1)	
Anxiety, *n* (%)	34 (40.5)	18 (23.1)	.005**	52 (41.9)	26 (23.2)	.003**
Depression, *n* (%)	21 (25.0)	4 (5.1)	.002**	21 (16.9)	3 (2.7)	.001**
Self-reported sexual dysfunction, *n* (%)	8 (9.5)	0	.007**	3 (2.4)	12 (9.7)	.017*

*p < 0.05, **p < 0.01.

In the female cohort, 34 (40.5%) IBD patients and 18 (23.1%) healthy controls were designated to have anxiety (*p* < 0.01), while 21 (25.0%) IBD patients and 4 (5.1%) healthy controls were designated as having depression (*p* < 0.01). In males, 52 (41.9%) IBD patients and 26 (23.2%) healthy controls were reported to have anxiety (*p* < 0.01), while 21 (16.9%) IBD patients and 3 (2.7%) healthy controls were considered to have depression (*p* < 0.01) (**Table 1**).

Characteristics of the 208 IBD patients included in this study are presented in **Table 2**. In females with IBD, 54 (64.3%) had CD, 30 (35.7%) had UC and 44 (52.4%) had active disease. In men, 79 (63.7%) had CD, 45 (36.3%) had UC and 69 (55.6%) had active disease.

**Table 2:** Characteristics of the IBD population.

	Female patients *n* = 84	Male patients *n* = 124
CD, *n* (%)	54 (64.3)	79 (63.7)
UC, *n* (%)	30 (35.7)	45 (36.3)
Median CDAI (IQR)	180 (87.3-280.8)	150 (75.5-185.5)
Median Mayo score (IQR)	5 (3-10.5)	7.5 (4-10.3)
Active disease, *n* (%)		
Mild disease course^a^	13 (15.5)	34 (27.4)
Moderate disease course^b^	23 (27.4)	26 (21.0)
Severe disease course^c^	8 (9.5)	9 (7.3)
Median disease duration (IQR), years	5 (2-8)	4 (2-7)
Active perianal disease, *n* (%)	21 (25.0)	14 (11.3)
Previous surgery, *n* (%)	30 (35.7)	46 (37.1)
Presence of a stoma, n (%)	4 (4.8)	3 (2.4)
5-ASA, current use, n (%)	40 (47.6)	60 (48.4)
Immunosuppressant, current use, n (%)	11 (13.1)	10 (8.1)
Biological therapy, current use, n (%)	45 (53.6)	66 (53.2)
Corticosteroids, current use, n (%)	8 (9.5)	10 (8.1)
Psychotropic medication, current use, *n* (%)	2 (2.4)	8 (6.5)

CD, Crohn’s disease; CDAI, Crohn’s disease activity index; UC, ulcerative colitis; IBD, inflammatory bowel disease; IQR, interquartile range; 5-ASA, 5-Aminosalicylic acid.

a Defined as CDAI score is 150-220 or Mayo score is 3-5.

b Defined as CDAI score is 220-450 or Mayo score is 6-10.

c Defined as CDAI score > 450 or Mayo score is 11-12.

### Sexual Dysfunction in Female IBD Patients

**Figure 1:**
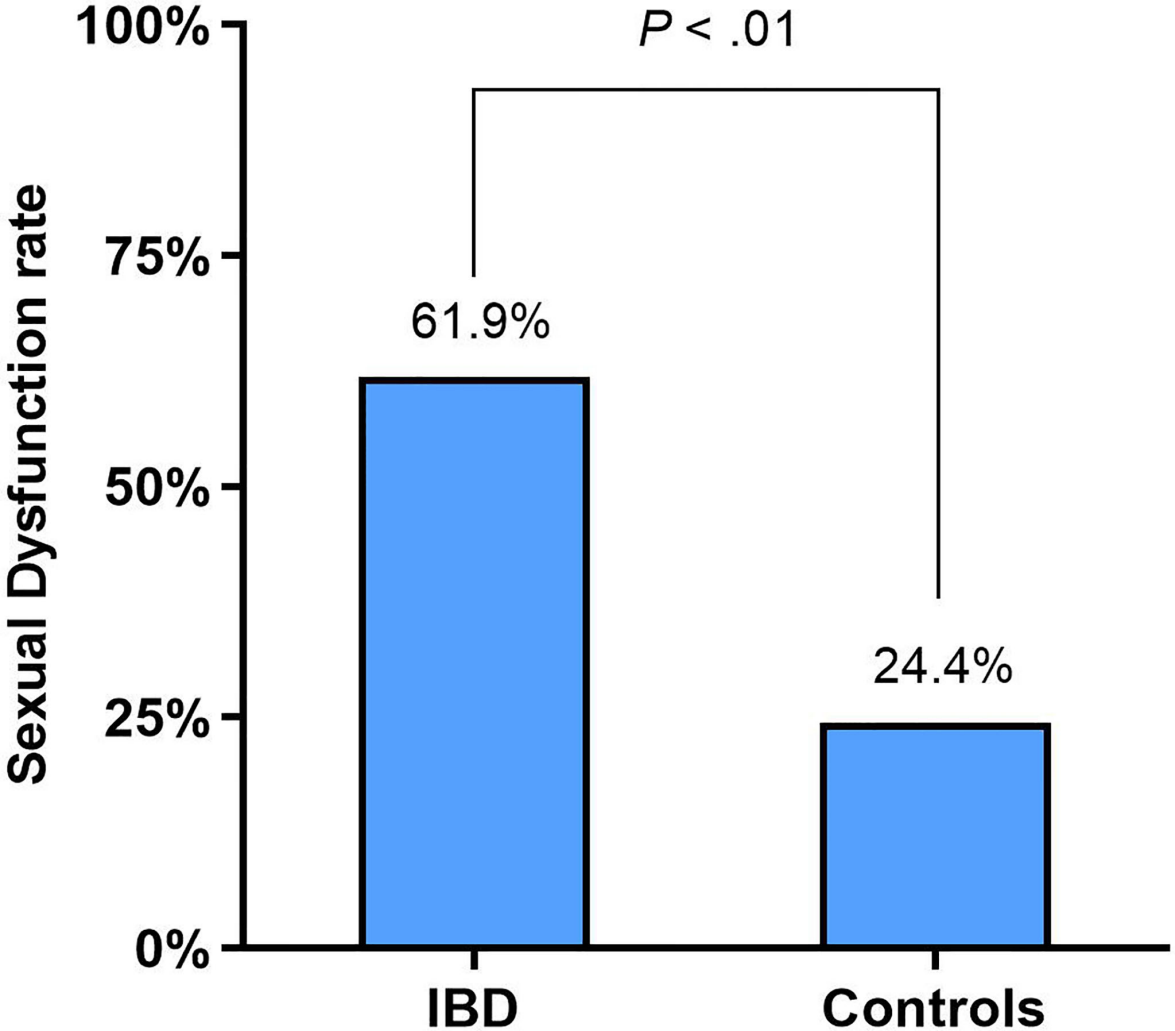
Sexual function of IBD female patients compared with healthy controls according to FSFI.

In women, SD was identified in 61.9% (52/84) of the IBD patients and 24.4% (19/78) of the healthy control individuals (*p* < 0.01) (**Figure 1**). The rate of SD was not different between CD and UC patients: 61.1% (33/54) and 63.3% (19/30), respectively (*p* = 0.841). The prevalence of SD in patients with CD and UC was significantly higher than that observed in the control group (all *p* < 0.01). The proportion of SD in IBD patients with the active disease was significantly higher than that of those patients in remission: 74.4% (32/43) and 48.8% (20/41), respectively (*p* = 0.016). The prevalence of SD in active and remission IBD patients was also significantly higher than that observed in the control patients (all *p* < 0.01).

The prevalence of SD in mild, moderate, and severe female IBD patients was 61.5%, 73.9%, and 87.5%, respectively, which were all significantly higher than in the control group (all *p* < 0.01). The prevalence of SD was higher in moderate and severe active IBD patients than in IBD-remission patients (all *p* < 0.05). Additionally, there was no significant difference between mildly active IBD patients and IBD-remission patients (*p* = 0.701). However, there was a significant difference in the prevalence between mildly active IBD patients and moderate and severe patients (all *p* < 0.05), as well as between moderately active IBD patients and severe IBD patients (*p* < 0.05). As the disease activity increased, the prevalence of SD in female IBD patients increased (all *p* < 0.05) (**Table 3**).

**Table 3:** Prevalence and subgroup analysis of sexual dysfunction in IBD patients.

	Women, *n* (%)	Men, *n* (%)
IBD	52 (61.9)^**^	54 (43.5)^**^
CD	33 (61.1)^**^	35 (44.3)^**^
UC	19 (63.3)^**^	19 (42.4)^**^
Disease Staging		
In remission	19 (47.5)^**^	17 (30.9)^**^
In active disease	32 (75.0)^**,ξ^	36 (53.6)^**,ξ^
Active disease index		
Mildly active disease	8 (61.5)^**^	16 (47.1)^**^
Moderately active disease	17 (73.9)^**,ξ^	15 (57.7)^**,ξξ^
Severely active disease	7 (87.5)^**,ξ^	5 (66.7)^**,ξξ^
Healthy controls	20 (24.4)	14 (12.5)

^*^P < 0.05, ^**^P < 0.01, vs. healthy controls; ^ξ^ P < 0.05, ^ξξ^ P < 0.01, vs. in remission.

CD, Crohn’s disease; IBD, inflammatory bowel disease; UC, ulcerative colitis.

When compared to age-matched control pairs, regarding sexual function from each domain of FSFI score for women (**Table 4**), IBD patients scored significantly lower than controls in sexual desire, arousal, lubrication, orgasm and global satisfaction (*p* < 0.01, *p* < 0.01, *p* < 0.01, *p* < 0.01, and *p* = 0.02, respectively). Pain scores in IBD patients were not different from controls (*p* = 0.61). The total FSFI score in IBD patients was significantly lower than controls (*p* < 0.01).

**Table 4:** Age-matched comparison of IBD patients and healthy controls for FSFI or IIEF-5.

	IBD patients Mean (*n*)	Healthy controls Mean (*n*)	*P*
FSFI			
Desire	3.0 (79)	4.0 (78)	.000**
Arousal	3.6 (79)	4.5 (78)	.000**
Lubrication	4.4 (79)	4.9 (78)	.000**
Orgasm	4.1 (79)	4.8 (78)	.000**
Global satisfaction	4.2 (79)	4.5 (78)	.019*
Pain	4.6 (79)	4.6 (78)	.612
Total score	23.8 (79)	27.2 (78)	.000**
IIEF-5			
Total score	20.4 (119)	22.2 (112)	.002**

Mean score, number of matched individuals, paired t-test p-value. *P <.05, **P <.01.

FSFI, female sexual function index; IBD, inflammatory bowel disease; IIEF-5, a simplified version of the international index of erectile function.

### Erectile Dysfunction in Male IBD Patients

**Figure 2:**
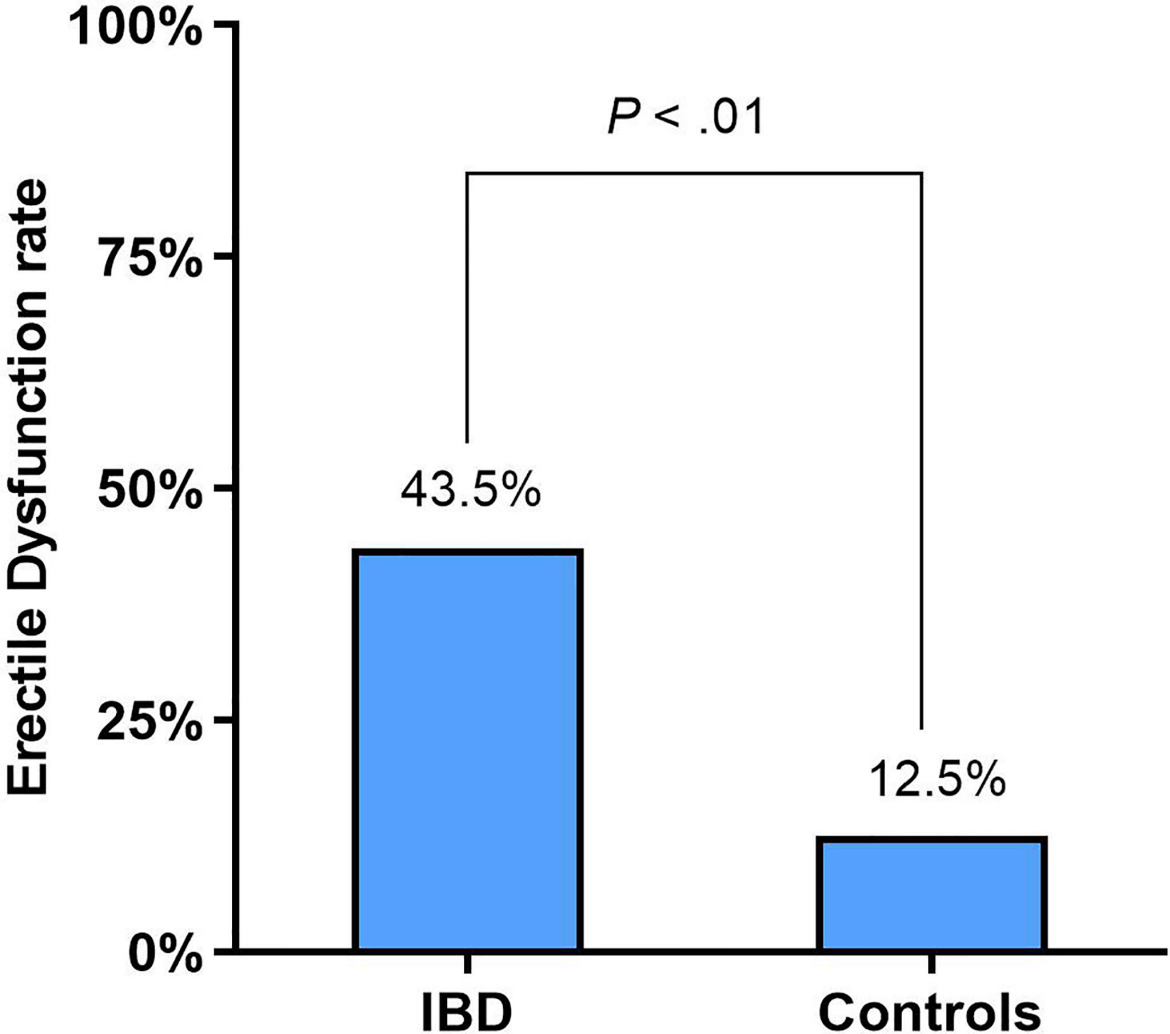
Erectile function of IBD male patients compared with healthy controls according to IIEF-5.

In men, ED was identified in 43.5% (54/124) of the IBD patients and 12.5% (14/112) of the healthy controls (*p* < 0.01) (**Figure 2**). The rate of ED was not different between CD and UC patients: 44.3% (35/79) and 42.4% (19/45), respectively (*p* = 0.822). However, The prevalence of ED in CD and UC patients was significantly higher than in the control group (all *p* < 0.01). The rate of ED in IBD patients with the active disease was significantly higher than in patients with IBD-remission: 53.6% (37/69) and 30.9% (17/55), respectively (*p* = 0.011), which were significantly higher than healthy controls (all *p* < 0.01).

The prevalence of ED in mild, moderate, and severe male IBD patients was 47.1%, 57.7%, and 66.7%, respectively, which are all significantly higher than reported in the control group (all *p* < 0.01). As the disease activity increased, the prevalence of ED in male IBD patients increased (all *p* < 0.05) (**Table 3**). The prevalence of ED was higher in moderate and severe IBD patients as compared to the IBD-remission patients (all *p* < 0.01), and there was no significant difference between mildly active and IBD-remission patients (*p* = 0.125). However, there was a significant difference in the prevalence between mildly active IBD patients and moderate and severe IBD patients (all *p* < 0.05), and between moderately active IBD patients and severe IBD patients (*p* < 0.05).

When compared to age-matched pairs, the total score of IIEF-5 in IBD patients was significantly lower compared to healthy controls (*p* < 0.05) (**Table 4**). In terms of ED severity, No severe ED in neither the IBD patients nor the healthy controls. The prevalence of mild, mild-moderate and moderate ED in IBD patients was higher than that in healthy controls, with statistically significant differences (all *p* < 0.01) (**Table 5**).

**Table 5:** Comparison of IBD patients and healthy controls for IIEF-5.

	IBD Patients, *n* (*%*)	Healthy controls, *n* (*%*)	*P*
Non-ED	70 (56.5)	98 (87.5)	
Mild ED^a^	34 (27.4)	10 (8.9)	<0.01**
Mild-moderate ED^b^	14 (11.3)	3 (2.7)	0.001**
Moderate ED^c^	6 (4.8)	1 (0.9)	0.044*
Severe ED^d^	–	–	
Erectile dysfunction	54 (43.5)	14 (12.5)	<0.01**

Number of individuals, Chi-squared p-value. *p < 0.05, **P < 0.01.

IBD, inflammatory bowel disease; IIEF-5, a simplified version of the international index of erectile function; ED, erectile dysfunction.

a Defined as IIEF-5 score is 17-21.

b Defined as IIEF-5 score is 12-16.

c Defined as IIEF-5 score is 8-11.

d Defined as IIEF-5 score is 5-7.

### Factors Associated With SD or ED in IBD Patients

In women, disease duration, disease activity, active perianal disease, anxiety, and depression were associated with SD in the univariate analysis. In multivariate analysis, anxiety (OR, 3.092; 95%CI: 1.033-9.252, *p* = 0.044) and active perianal disease (OR, 4.481; 95%CI: 1.055-19.029, *p* = 0.042) were independently associated with SD.

In men, age, disease activity, active perianal disease, and depression were associated with ED in the univariate analysis. In multivariate analysis, age (OR, 1.050; 95%CI: 1.007-1.095, *p* = 0.022), depression (OR, 5.763; 95%CI: 1.864-17.821, *p* = .002) and active perianal disease (OR, 7.117; 95%CI: 1.747-28.983, *p* = 0.006) were independently associated with ED.

## Discussion

SD in IBD is often ignored by physicians, and not enough attention is paid to the patient. Due to various factors, such as social and religious beliefs, it is difficult to actively talk about sexual function with patients. Therefore, SD has become a “blind spot” in the medical field, which is an important factor affecting the physical and mental health of patients. Sometimes, it aggravates the psychosomatic disorder of patients, making the treatment of IBD more difficult and can seriously affect the quality of life of many IBD patients (16, 17).

This study found that SD in female IBD patients was 61.9% and ED was 43.5% in male IBD patients, both of which were significantly higher than that reported in the control groups. This suggests that IBD patients are more prone to SD than healthy individuals. It was found that the prevalence of SD was 40%-71% and 30%-55% in male and female IBD patients, respectively (16–21). One interesting finding was that there is a high prevalence of SD in IBD-active female patients. The reason is unclear, but we hypothesize that it may be due to the fact that women are more susceptible to abdominal pain, diarrhea, and other uncomfortable abdominal symptoms when the disease is active (19).

We found that IBD subtype and SD are unrelated. Further subgroup analysis of disease activity showed that there was no significant difference in the prevalence of SD between patients with mild IBD activity and those in remission, regardless of gender, indicating that mild activity of IBD had little influence on the sexual function of patients. In addition, patients with moderate to severe activity were more likely to develop SD than those in remission, and SD increased with the increase of disease activity. Besides, our study found that IBD had the most significant impact on female sexual desire and arousal.

Studies have found that IBD disease activity is a risk factor for SD (19, 20), which is consistent with our findings. Increased disease activity can lead to low libido, especially in female patients. Active patients have more lubrication problems and dyspareunia. Worries about discomfort such as abdominal pain and diarrhea make the pelvic floor muscles tense, which may lead to painful intercourse and lubrication problems due to fear of pain (19, 20). In women, disease duration is a risk factor for SD. IBD patients with long disease duration (> 3 years) were more likely to experience decreased libido (about 2.59 times) (7). However, sexual function was relatively better when the disease lasted longer (21). The reason may be that risk factors affecting sexual function increase and may even decrease over time. In the course of the disease, symptoms are constantly changing, and it is challenging to control confounding factors other than disease duration. Therefore, the effect of disease duration on the sexual function of IBD cannot be accurately assessed.

An important finding of this study was that active perianal disease was an independent risk factor for SD. Moody *et al.* reported that CD patients, especially those with anal fistula, are prone to dyspareunia or even no sexual activity (22). Kappelman *et al.* reported low sexual desire and satisfaction in the cohort of patients with the active perirectal disease (23). Horst *et al.* analyzed perianal disease in IBD patients treated with a combination of medication and surgery and found that 65% of patients had moderate to severe SD at baseline, with only 11% reporting moderate SD after 48 weeks of treatment (24). These results suggest that active perianal disease can impair sexual function in IBD, and a cure can alleviate SD.

Our study also found that anxiety and depression were independent risk factors for SD in women and ED in men. Due to mental and physical discomfort, anxiety and depressive symptoms have a higher prevalence in IBD patients, with anxiety being more common in women (25). In previous studies, anxiety (6, 17) and depression (7, 18–20) have been associated with SD in IBD patients, which is consistent with our research. In addition, we found that antipsychotics were not a risk factor for SD in IBD patients, and Riviere *et al.* also reported this finding (16). Therefore, for IBD patients whose sexual function declines due to psychological problems, antipsychotic drugs can be used when necessary.

Age was an independent risk factor for ED in men in our study. Aging is a well-known risk factor for SD in both sexes. It was found that SD was associated with older age in IBD patients (17). As men age, ED increases due to hypogonadism (26). It has been reported that hypogonadism is also a leading factor of ED in IBD patients (8) This may be due to the inflammatory response of IBD, leading to hypogonadism and subsequently SD. In addition, endothelial dysfunction may contribute to the development of ED in patients with IBD. IBD can cause microvascular endothelial dysfunction, which is characterized by the loss of nitric oxide (NO)-dependent dilation, contributing to reduced perfusion, poor wound healing, and persistent chronic inflammation (27). In the meta-analysis of Zhao *et al.* (28), subgroup analysis based on average individual age showed that younger IBD patients (male: < 50 years old; female: < 40 years old) had a significantly increased incidence of SD, but no positive correlation was found in older men and women, which may be related to the fact that the onset of IBD is mostly in young and middle-aged adults (29). In conclusion, IBD can lead to impaired sexual function. Age may be a risk factor for SD independent of IBD, or age and IBD may act together to cause SD.

This study found no association between IBD-related surgical history and SD, which may be due to the different surgical methods, operators, and postoperative recovery of different patients, resulting in different effects of surgery on sexual function. We also found no association between IBD-related medications and SD. A survey of 347 IBD patients in Australia found that more than 60% of respondents were not concerned about the effect of medication on libido or frequency of sexual activity. However, there are still a small number of patients (9.7%) who deliberately do not use drugs because they believe that drugs have negative effects on sexual function (30). Therefore, education on drug use should be conducted for IBD patients to increase their medication compliance.

Ours is the first study from China evaluating SD in women and ED in men who suffer from IBD. Our research was conducted by trained investigators who issued questionnaires and guided patients to complete them. Since there can be valuable differences in the geographical and cultural aspects, we believe that the study will bring new insights to this field.

However, some limitations have been encountered in this study. First, only IIEF-5 was used to evaluate the sexual function of male patients, which paid too much attention to the ED of IBD patients and ignored other aspects of SD, such as premature ejaculation, orgasm, and global satisfaction. Second, the questionnaire was not specific for IBD, and there were no specific questions about the effects of surgery, perianal disease, or treatment on sexual function. Third, since the lack of sexual partners will affect the questionnaire scores on sexual function, patients without sexual partners were not included in the study. The questionnaire we used is more suitable for patients with sexual partners. Particularly FSFI, women who have not had sex in the past four weeks score very low, which would be categorized as SD. However, it is possible that some patients do not have a sexual partner because of their sexual problems. In this case, it is possible to underestimate problems in the patient population.

## Conclusions

62% of women with IBD have SD, and 44% of men with IBD have ED. Active perianal disease, anxiety, and depression are independent risk factors for SD in IBD patients. Clinicians should evaluate the sexual function of IBD patients and perform the necessary treatment to improve the quality of life of patients suffering from IBD.

## Data Availability Statement

The original contributions presented in the study are included in the article/**Supplementary Material**. Further inquiries can be directed to the corresponding author.

## Ethics Statement

The studies involving human participants were reviewed and approved by Ethics Committee on Biomedical Research, West China Hospital of Sichuan University. The patients/participants provided their written informed consent to participate in this study.

## Author Contributions

JZ and JN have contributed equally to this work. HG, JZ and JN had full access to all the data in the study and take responsibility for the integrity of the data and the accuracy of the data analysis. Study concept and design, all authors. Acquisition of data, JZ, JN, MZ, and QZ. Analysis and interpretation of data, all authors. Drafting of the manuscript, all authors. Critical revision of the manuscript for important intellectual content, all authors. Statistical analysis, JZ and JN. Obtaining funding, HG. Supervision, HG. All authors contributed to the article and approved the submitted version.

## Funding

This study was funded by the National Natural Science Foundation of China (NO. 81470826), the Science Foundation from Science and Technology Department of Sichuan Province (NO. 2019YFS0262), and 1.3.5 project for disciplines of excellence, West China Hospital, Sichuan University (NO. ZYGD18023).

## Conflict of Interest

The authors declare that the research was conducted in the absence of any commercial or financial relationships that could be construed as a potential conflict of interest.

## Publisher’s Note

All claims expressed in this article are solely those of the authors and do not necessarily represent those of their affiliated organizations, or those of the publisher, the editors and the reviewers. Any product that may be evaluated in this article, or claim that may be made by its manufacturer, is not guaranteed or endorsed by the publisher.
