# Supplementary material for: Prevalence and Associated Factors of Sexual Dysfunction in Patients With Inflammatory Bowel Disease
Source: Front Endocrinol (Lausanne). 2022 Apr 22;13:881485. doi: 10.3389/fendo.2022.881485 (PMC9094619; doi:10.3389/fendo.2022.881485)
Supplement: Supplementary file 1 [file Table_1.docx]

Supplementary Table 1. Comparison of participants and patients declining to participate

|  | Participants  *n* = 208 | Non-participants  *n* = 20 | *P* |
| --- | --- | --- | --- |
| Women, *n* (%) | 84 (40.4) | 8 (40.0) | .973 |
| Median age at inclusion (IQR), years | 33 (28-44.8) | 31(26-40) | .701 |
| IBD subtype, *n* (%) |  |  | .841 |
| CD | 133 (63.9) | 13 (65.0) |  |
| UC | 75 (36.1) | 7 (35.0) |  |
| Median CDAI (IQR) | 180 (87.3-280.8) | 150.5 (85.1-209.6) | .791 |
| Median Mayo score (IQR) | 5 (3-10.5) | 9.5 (5-11) | .620 |
| Active disease, *n* (%) |  |  | .258 |
| Mild disease course^a^ | 47 (22.6) | 6 (30.0) |  |
| Moderate disease course^b^ | 49 (23.6) | 2 (10.0) |  |
| Severe disease course^c^ | 17 (8.2) | 4 (20.0) |  |
| Median disease duration (IQR), years | 4 (2-7.8) | 5 (2-9.8) | .583 |
| Active perianal disease, *n* (%) | 35 (16.8) | 3 (15.0) | .177 |
| Previous surgery, *n* (%) | 76 (36.5) | 7 (35.0) | .200 |
| Presence of a stoma, n (%) | 7 (3.4) | 0 | 1.00 |
| 5-ASA, current use, n (%) | 100 (48.1) | 5 (25.0) | .431 |
| Immunosuppressant, current use, n (%) | 21 (10.1) | 2 (10.0) | 1.00 |
| Biological therapy, current use, n (%) | 111 (53.4) | 9 (45.0) | .474 |
| Corticosteroids, current use, n (%) | 18 (8.7) | 4 (20.0) | .111 |
| Psychotropic medication, current use, *n* (%) | 10 (4.8) | 0 | .606 |

CD, Crohn's disease; UC, Ulcerative colitis; IQR, interquartile range; CDAI, Crohn's disease activity index. ^a^Defined as CDAI score is 150-220 or Mayo score is 3-5. ^b^Defined as CDAI score is 220-450 or Mayo score is 6-10. ^c^Defined as CDAI score > 450 or Mayo score is 11-12.
