# Supplementary material for: Prevalence and Associated Factors of Sexual Dysfunction in Patients With Inflammatory Bowel Disease
Source: Front Endocrinol (Lausanne). 2022 Apr 22;13:881485. doi: 10.3389/fendo.2022.881485 (PMC9094619; doi:10.3389/fendo.2022.881485)
Supplement: Supplementary file 2 [file Table_2.doc]

Supplementary Table 2. Factors associated with erectile dysfunction in IBD male patients and sexual dysfunction in IBD female patients, in univariate and multivariate logistic regression

| Variables | Univariate analysis | | | | Multivariate analysis | | | |
| --- | --- | --- | --- | --- | --- | --- | --- | --- |
|  | Male patients IBD | | Female patients IBD | | Male patients IBD | | Female patients IBD | |
|  | OR (95% CI) | *P* | OR (95% CI) | *P* | OR (95% CI) | *P* | OR (95% CI) | *P* |
| Age  BMI  Education  Relationship status  Active smoking  Active Drinking  Profession  Address | 1.042 (1.007-1.079) | .019*  *us*  0.096°  *us*  *us*  *us*  *us*  *us* |  | *us*  *us*  *us*  *us*  *us*  *us*  *us*  *us* | 1.050 (1.007-1.095)  - | .022*  - |  |  |
| IBD subtype  Disease duration  Active disease ^a^  Active disease index^b^  Prior surgery  Stoma present at inclusion  Active perianal disease  History of perianal disease  IBD-related comorbidities^c^  Joint pain  Extraintestinal manifestations^d^  5-ASA, current use | 2.242 (1.075-4.677)  3.750 (1.106-12.710) | *us*  *us*  .031*  *us*  *us*  *us*  .034*  *us*  0.111°  *us*  *us*  *us* | 1.184 (1.032-1.359)  2.647 (1.068-6.560)  4.296 (1.140-16.189)  6.55 (1.76-24.30) | *us*  .016*  .036*  *us*  *us*  *us*  .031*  *us*  *us*  0.151°  *us*  *us* | -  7.117 (1.747-28.983)  - | -  .006*  - | -  -  4.481 (1.055-19.029)  - | -  -  .042*  - |
| Corticosteroids, current use  Thiopurines, current use  Biological therapy, current use  Psychotropic medication, current use  Anxiety  Depression | 4.103 (1.469-11.458) | *us*  *us*  *us*  *us*  *us*  .007* | 3.000(1.140-7.893)  3.400 (1.027-11.257) | *us*  *us*  *us*  *us*  .026*  .045* | 5.763 (1.864-17.821) | .002* | 3.092 (1.033-9.252)  - | .044*  - |

BMI, Body Mass Index; IBD, inflammatory bowel disease; OR, odds ratio; 95% CI, 95% confidence interval; us, *P* > 0.20; °, 0.05 < *P* < 0.20, factors included in the multivariate model. **P* < 0.05.

^a^ Defined as CDAI score >= 150 for Crohn's disease (CD), Defined as Mayo score > 2 for Ulcerative Colitis (UC). ^b^ Mild disease course, defined as CDAI score is 150-220 or Mayo score is 3-5; Moderate disease course, defined as CDAI score is 220-450 or Mayo score is 6-10; Severe disease course, defined as CDAI score > 450 or Mayo score is 11-12. ^c^ Including bowel obstruction, bowel fistula, and peritoneal abscess. ^d^ Including erythema nodosum, gangrenous pyoderma, scleritis, uveitis, and primary sclerosing cholangitis.
